# Sex based disparities in glycemic control and hospitalization outcomes of medical patients with diabetes mellitus—a historical cohort study

**DOI:** 10.1007/s11739-025-04170-4

**Published:** 2025-11-01

**Authors:** Ronit Koren, Matan Elkan, Arielle Barouch, Tomer Ziv-Baran

**Affiliations:** 1 Department of Internal Medicine A, Shamir Medical Center, 7030000 Zerifin, Israel; 2 Gray School of Medicine, Gray Faculty of Medical & Health Sciences, Tel Aviv University, Tel Aviv, Israel; 3 Department of Epidemiology and Preventive Medicine, School of Public Health, Gray Faculty of Medical & Health Sciences, Tel Aviv University, Tel Aviv, Israel

**Keywords:** Diabetes Mellitus, Sex, Hospitalization, Glycemic control, Chronic treatment patterns

## Abstract

While sex differences in type 2 diabetes mellitus (T2DM) are well-documented in outpatient settings, data on inpatient disparities remain limited. This study examines the relationship between sex and glycemic control, chronic treatment patterns, and hospitalization outcomes in patients with T2DM. A historical cohort of 5,133 adult T2DM patients hospitalized for > 24 h at a tertiary Israeli medical center in 2023 was analyzed. Outcomes included hypoglycemia, hyperglycemia, length of stay, 30-day readmission, and mortality. Propensity score matching and multivariable regression were applied to adjust for confounding variables. Men were more frequently treated with chronic aspirin therapy than women (35.8% vs. 29.4%; *p* < 0.001), a difference that remained significant after multivariable adjustment (aOR 1.31; 95% CI: 1.13–1.52; *p* < 0.001). Male sex was also associated with higher rates of hyperglycemia (76.4% vs. 73.3%; *p* = 0.009) and severe hyperglycemia (48.6% vs. 43.7%; *p* < 0.001), with these associations persisting after adjustment (hyperglycemia: aOR 1.23; 95% CI: 1.03–1.46; *p* = 0.02; severe hyperglycemia: aOR 1.28; 95% CI: 1.09–1.49; *p* = 0.002) and confirmed in propensity score-matched analyses. One-month rehospitalization rates were also higher in men (12.7% vs. 11.1%; *p* = 0.088), reaching statistical significance after multivariable adjustment (aOR 1.27; 95% CI: 1.03–1.55; *p* = 0.02). Sex-based disparities persist in inpatient glycemic control and chronic treatment patterns. Men are more prone to hyperglycemia and rehospitalization, while women are less likely to receive guideline-recommended cardioprotective therapies. A sex-specific approach may improve inpatient diabetes management and long-term outcomes.

**Supplementary Information:**

The online version contains supplementary material available at 10.1007/s11739-025-04170-4.

## Introduction

Sex plays a crucial role in precision medicine, influencing nearly all aspects of disease pathophysiology, including mechanism, prevalence, progression, treatment response, and prognosis [1]. In diabetes, well-documented sex differences exist in metabolic regulation and cardiovascular risk [2]. Men have a higher prevalence of type 2 diabetes mellitus (T2DM) in young and middle-aged populations, whereas women exhibit a higher prevalence of undiagnosed diabetes and an increased diabetes risk after the age of 70 [3, 4]. Men more frequently present with impaired fasting glucose, while women more commonly exhibit impaired glucose tolerance [5].

Disparities in clinical care and treatment patterns parallel these sex-based biological differences. In a large outpatient cohort, diabetes care was of poorer quality in women compared with men, including in achieving glucose and lipid targets and diabetic foot monitoring [6]. Furthermore, sex-based disparities exist in medication prescriptions. It seems that the perception of cardiovascular risk is lower for women; hence, preventive measures are recommended less often [7]. For example, men with cardiovascular disease or heart failure (HF) receive sodium-glucose co-transporter-2 (SGLT2) inhibitors more often and at an earlier stage than women [8, 9]. Similarly, women are less frequently prescribed cardioprotective medications such as statins, aspirin, and angiotensin-converting enzyme (ACE) inhibitors [10, 11], and anti-diabetic guideline directed medical therapy [12]. Patient adherence to prescribed therapies may represent an additional factor contributing to sex-based differences in care. Large cohorts demonstrate increase in cardiovascular morbidity and mortality in women with diabetes (but not with prediabetes) compared with men [13]. Differences in care and lifestyle may only partly explained the higher incidence of coronary heart disease and cardiovascular mortality in diabetic women [14, 15].

Despite well-established sex differences in outpatient diabetes care, data on disparities among hospitalized patients with diabetes remain limited. Compared to the general population, individuals with diabetes face an increased risk of hospitalization [16], and readmission [17]. Sex-based differences have been reported in hospitalization and readmission rates, causes of admission, risk of hypoglycemia, and discharge destination [18, 19].

Although sex disparities in glycemic control have been extensively described in the outpatient setting, little is known about sex-based differences among hospitalized patients with T2DM. The inpatient environment presents unique challenges for glucose management due to acute stress responses, altered nutritional intake, intensive monitoring, and different treatment algorithms compared to the outpatient setting. Importantly, both hyperglycemia and hypoglycemia during hospitalization have been shown to adversely impact short- and long-term outcomes.

[20] Most guidelines recommend initiating insulin therapy in critically ill and non-critically ill patients with glucose levels >180 mg/dL, aiming for glycemic targets of 100–180 mg/dL [21]. However, data on sex differences in inpatient glycemic control are scarce, with limited evidence such as a small study reporting greater insulin resistance in critically ill women [22].

This study aims to evaluate sex-based differences in glycemic control, hospital length of stay, and short- and long-term mortality among patients with T2DM, adjusting for baseline comorbidities and clinical characteristics. Addressing these gaps is critical, as identifying sex-specific disparities in may inform tailored management strategies and ultimately improve outcomes for both sexes.

## Methods

### Study design and participants

This was a historical cohort study including all consecutive adult patients (aged >18 years) with type 2 diabetes mellitus (T2DM) who were hospitalized for more than 24 h in medical wards at Shamir Medical Center (SMC) between January 1, 2023, and December 31, 2023.

SMC is a 904-bed university-affiliated tertiary medical center located in Israel’s central region and serves urban and rural populations. SMC comprises 7 internal medicine departments with a total of 269 beds. Hospitalization outcomes were assessed through February 2024. Patients were excluded if they were admitted for acute diabetes-related complications (diabetic ketoacidosis or hyperosmolar state), were pregnant, or had type 1 diabetes mellitus.

### Data collection

Data was accessed via the Israeli Ministry of Health’s Kineret Platform. Kineret is a cloud-based service that facilitates secure, anonymized analysis of electronic health records structured within the Observational Medical Outcomes Partnership (OMOP) Common Data Model [23]. The cohort was designed and characterized using ATLAS [24], an open-source tool developed by the Observational Health Data Sciences and Informatics (OHDSI) community [25]. Ethical approval was obtained from the local institutional ethics committee before study initiation (approval number 0224-24ASF).

### Study variables, measurements, and definitions

Comorbidities were identified using International Classification of Diseases (ICD-9-CM) codes, while medications were classified according to the Anatomical Therapeutic Chemical (ATC) system (Supplementary Tables S1 and S2).

Laboratory indices, including hemoglobin, albumin, creatinine, electrolytes, and C-reactive protein (CRP), were collected from the first available post-admission results up to 24 hours from arrival. Glomerular filtration rate (GFR) was calculated using the “CKD-EPI” equation. Acute kidney injury (AKI) was defined as an increase in serum creatinine of ≥0.3 mg/dL during hospitalization.

We also collected data on chronic cardioprotective treatment such as the use of antiplatelets (aspirin, clopidogrel), ACE inhibitors, angiotensin receptor blockers (ARBs), statins, and SGLT2 inhibitors before hospitalization.

The primary outcomes were based on 4 levels of glycemic control during hospitalization: hypoglycemia (≤70 mg/dL), severe hypoglycemia (≤54 mg/dL), hyperglycemia (>180 mg/dL), and severe hyperglycemia (>250 mg/dL). We analyzed the percentage of all measurements in range of 100–180 mg/dL during hospitalization. Data were derived from blood tests and point-of-care glucose monitoring.

Additional outcomes included length of stay, rehospitalization within one month, and all-cause mortality during hospitalization and within 30-days since hospital admission. Mortality data were obtained from the Israeli Ministry of Interior’s national registry.

### Sample size

A two-group design was employed to investigate whether the proportions of the studied outcome differ between men and women. The sample size calculation was made using a two-sided, two-sample Z-test, with a Type I error rate (α) of 0.01, a power of 90%, and an equal number of patients in each group. To detect a small effect (effect size = 0.2) of sex on the studied outcomes, the number of subjects needed in each group was 744.

### Statistics

Categorical variables were described as frequencies and percentages. Continuous variables were evaluated for normal distribution using histograms and reported as means and standard deviations or as medians and interquartile ranges (IQR). The chi-square test was used to compare categorical variables between the sex groups, and the independent samples t-test and Mann-Whitney tests were applied to compare continuous variables. Multivariable logistic regression was applied to evaluate the association between sex and the studied outcomes while controlling for possible known confounders. Each regression contained two blocks. In the first block, sex and age were forced into the regression. In the second block, the following variables were considered for inclusion using the forward selection method (the Wald test was used and *p* < 0.05 was the criterion for inclusion): Chronic Obstructive Pulmonary disease (COPD)/asthma, pulmonary embolism, liver disease, rheumatic disease, chronic infection, hypertension (HTN), HF, ischemic heart Disease, atrial fibrillation/flutter, cardiac arrhythmias, peripheral artery disease (PVD), dyslipidemia, past stroke\transient ischemic attack (TIA), dementia, malignancy, systemic steroids, white blood cells (WBC), lymphocytes, neutrophils, hemoglobin, platelets, C-reactive protein (CRP), albumin, estimated GFR (eGFR), calcium, corrected calcium level, potassium, sodium, diastolic blood pressure (BP), systolic BP, pulse, temperature, glucose levels, high density lipoprotein (HDL), triglycerides, low density lipoprotein (LDL), and body mass index (BMI).

The two sex groups were matched according to the probability of a patient being a male. The probability (propensity score) was calculated using a logistic regression model. The following parameters were used to calculate the propensity score: age, BMI, systemic steroids, COPD/asthma, liver disease, chronic infection, HTN, HF, ischemic heart disease (IHD), atrial fibrillation flutter, acute arrhythmia, PVD, dyslipidemia, past stroke/TIA, dementia, and malignancy.

To strengthen our findings [26], propensity score matching was performed using 1:1 nearest neighbor matching with a 0.05 caliper width to balance covariates between groups. Standardized differences were calculated to compare the two sex groups, before and after matching. A standardized difference < 0.1 was considered a negligible difference, and a difference between 0.1 and 0.2 was considered a small difference (Supplementary Table S3). The matched groups were compared using the McNamar test for categorical variables, and the paired t-test or Wilcoxon test for the continuous variables.

All the statistical tests were two-sided, and *p* < 0.05 was considered statistically significant. Statistical analysis was performed using R (version 4.1.2, R Foundation for Statistical Computing, Austria, 2023).

## Results:

### Study population – unmatched cohort

Overall, 5133 patients met the criteria and were included in the study. Of them, 2,845 were men and 2,288 were women. Their demographic characteristics, comorbidities, and chronic medications are presented in Table 1. Before matching, women were older (median 77 vs. 74 years, *p* < 0.001) and had a higher BMI (median 28.1 vs. 27.3 kg/m^2^, *p* < 0.001). Women had higher rates of HTN (52.7% vs. 47.0%), while men had more IHD (26.6% vs. 13.6%), cerebrovascular disease (11.7% vs. 8.7%), CKD (17.2% vs. 14.1%), and PVD (5% vs. 1.8%), *p* < 0.001 for all comparisons. Men were more frequently treated with aspirin (35.8% vs. 29.5%, *p* < 0.001) and clopidogrel (11% vs. 7.2%, *p* < 0.001). Overall, few people were treated with GLP1 agonists or SGLT2 inhibitors. Men were more likely to receive SGLT2 inhibitors (7.8% vs. 5% *p* < 0.001) and long-acting insulin (40% vs. 35.4 %, *p* < 0.001). Women were more likely to be treated for HTN (44.7% vs. 41.5%, *p* = 0.024). There were no significant differences (*p* > 0.05) in the use of ACE inhibitors or ARBs.

**Table 1:** Baseline characteristics, before and after matching

	Unmatched cohort			Matched cohort		
	Men (*n* = 2845)	Women (*n* = 2288)	*P*-value	Men (*n* = 1755)	Women (*n* = 1755)	*P*-value
Age, years, median [IQR]	74 [66–81]	77 [70–85]	< 0.001	75 [68–82]	75 [68–83]	0.243
BMI, kg/m^2^, median [IQR]	27.3 [24.5–30.9]	28.1 [24.8–32.4]	< 0.001	27.7 [24.7–31.2]	27.7 [24.2–31.6]	0.981
Comorbidities						
HTN, n (%)	1337 (47.0%)	1206 (52.7%)	< 0.001	881 (50.2%)	882 (50.3%)	> 0.999
Dyslipidemia, n (%)	1331 (46.8%)	1118 (48.9%)	0.138	857 (48.8%)	839 (47.8%)	0.555
CHF, n (%)	280 (9.8%)	224 (9.8%)	0.951	159 (9.1%)	160 (9.1%)	> 0.999
IHD, n (%)	758 (26.6%)	312 (13.6%)	< 0.001	296 (16.9%)	277 (15.8%)	0.297
Atrial fibrillation/flutter, n (%)	369 (13.0%)	359 (15.7%)	0.005	230 (13.1%)	250 (14.2%)	0.358
Past CVA/TIA, n (%)	334 (11.7%)	198 (8.7%)	< 0.001	167 (9.5%)	161 (9.2%)	0.764
CKD, n (%)	488 (17.2%)	322 (14.1%)	0.003	286 (16.3%)	232 (13.2%)	0.012
COPD/Asthma, n (%)	300 (10.5%)	242 (10.6%)	0.970	183 (10.4%)	179 (10.2%)	0.866
Liver disease, n (%)	83 (2.9%)	99 (4.3%)	0.007	61 (3.5%)	63 (3.6%)	0.924
Connective tissue disease, n (%)	36 (1.3%)	110 (4.8%)	< 0.001	25 (1.4%)	85 (4.8%)	< 0.001
PVD, n (%)	143 (5.0%)	42 (1.8%)	< 0.001	43 (2.5%)	40 (2.3%)	0.812
Dementia, n (%)	51 (1.8%)	72 (3.1%)	0.002	40 (2.3%)	37 (2.1%)	0.82
Malignancy, n (%)	260 (9.1%)	218 (9.5%)	0.633	174 (9.9%)	169 (9.6%)	0.819
Charlson Index^b^, median [IQR]	5 [4–7]	5 [4–7]	< 0.001	5 [4–7]	5 [4–7]	0.201
Chronic Medications						
Metformin, n (%)	1257 (44.2%)	1043 (45.6%)	0.315	787 (44.8%)	814 (46.4%)	0.360
Long-acting Insulin, n (%)	379 (13.3%)	281 (12.3%)	0.268	229 (13.0%)	210 (12.0%)	0.332
Short-acting Insulin, n (%)	153 (5.4%)	116 (5.1%)	0.623	79 (4.5%)	91 (5.2%)	0.345
SU, n (%)	141 (5.0%)	87 (3.8%)	0.046	89 (5.1%)	70 (4.0%)	0.123
Non-SU, n (%)	151 (5.3%)	100 (4.4%)	0.122	96 (5.5%)	65 (3.7%)	0.012
DPP4, n (%)	154 (5.4%)	143 (6.2%)	0.202	95 (5.4%)	102 (5.8%)	0.608
GLP1 agonists, n (%)	147 (5.2%)	111 (4.9%)	0.607	100 (5.7%)	95 (5.4%)	0.761
SGLT2 inhibitor, n (%)	223 (7.8%)	115 (5.0%)	< 0.001	135 (7.7%)	97 (5.5%)	0.013
Any BP Medication n (%)^a^	1181 (41.5%)	1022 (44.7%)	0.023	734 (41.8%)	745 (42.5%)	0.707
Ace inhibitors/ARB	1310 (46.0%)	1091 (47.7%)	0.242	831 (47.4%)	814 (46.4%)	0.581
Plavix, n (%)	313 (11.0%)	165 (7.2%)	< 0.001	168 (9.6%)	136 (7.7%)	0.059
Aspirin, n (%)	1019 (35.8%)	674 (29.5%)	< 0.001	609 (34.7%)	515 (29.3%)	< 0.001
Statins, n (%)	1532 (53.8%)	1150 (50.3%)	0.011	931 (53.0%)	879 (50.1%)	0.085
Thiazolidinediones, n (%)	36 (1.3%)	31 (1.4%)	0.779	20 (1.1%)	27 (1.5%)	0.304
Diuretics, n (%)	404 (14.2%)	394 (17.2%)	0.003	253 (14.4%)	278 (15.8%)	0.239
Systemic Steroids, n(%)	271 (9.5%)	231 (10.1%)	0.494	182 (10.4%)	170 (9.7%)	0.500
Long-acting Insulin, n (%)	1137 (40.0%)	809 (35.4%)	< 0.001	697 (39.7%)	639 (36.4%)	0.044
Short-acting Insulin, n (%)	1066 (37.5%)	815 (35.6%)	0.172	657 (37.4%)	656 (37.4%)	0.972

ACE, angiotensin converting enzyme; ARB, angiotensin receptor blockers; BMI, body mass index; CHF, congestive heart failure; CKD, chronic kidney disease; COPD, chronic obstructive pulmonary disease; CVA, cerebrovascular accident; DPP4, Dipeptidyl peptidase-4; GLP-1, Glucagon-like peptide-1; HTN, hypertension, IHD, ischemic heart disease; PVD, peripheral vascular disease; SU, sulfonylurea; SGLT2, sodium-glucose transport protein 2; TIA, transient ischemic stroke

^a^Including- ACE inhibitors, ARB, thiazide diuretics, alpha antagonists, beta blockers

^b^Charlson index [39]

The cause of admission differed between men and women. Acute infection was slightly more common in men than in women (33.6% vs. 30.1%, p = 0.007). Chest pain and acute coronary syndrome (ACS) were more frequent in men (17.3 % vs. 12.8%, *p* < 0.001, and 32.9% vs. 18.4% *p* < 0.001, respectively), manifested in higher troponin levels in men (median 25 vs. 21 ng/L, *p* < 0.001). Women had higher rates of pulmonary embolism (2.1% vs 0.7% *p* < 0.001). LDL levels were higher in women (median 73 vs. 62mg/dL *p* < 0.001), which correlated with lower statin use in women (50.3% vs. 53.8%, *p* = 0.012). Men were also treated more often with SGLT2 inhibitors (7.8% vs. 5% *p* > 0.001). (Table 2)

**Table 2:** Hospitalization characteristics, before and after matching

	Unmatched cohort			Matched cohort		
	Men (*n* = 2845)	Women (*n* = 2288)	*P*-value	Men (*n* = 1755)	Women (*n* = 1755)	*P*-value
Cause of admission						
Acute infection, n (%)	956 (33.6%)	688 (30.1%)	0.007	585 (33.3%)	521 (29.7%)	0.019
Chest pain, n (%)	491 (17.3%)	294 (12.8%)	< 0.001	293 (16.7%)	241 (13.7%)	0.016
Dyspnea, n (%)	273 (9.6%)	282 (12.3%)	0.002	171 (9.7%)	205 (11.7%)	0.071
COPD/Asthma, n (%)	326 (11.5%)	261 (11.4%)	0.954	198 (11.3%)	193 (11.0%)	0.829
ADHF, n (%)	465 (16.3%)	386 (16.9%)	0.614	285 (16.2%)	286 (16.3%)	> 0.999
ACS, n (%)	936 (32.9%)	420 (18.4%)	< 0.001	430 (24.5%)	364 (20.7%)	0.002
Acute atrial fibrillation, n (%)	457 (16.1%)	442 (19.3%)	0.002	300 (17.1%)	308 (17.5%)	0.755
Acute TIA/CVA, n (%)	489 (17.2%)	332 (14.5%)	0.009	271 (15.4%)	248 (14.1%)	0.295
AKI^a^, n (%)	468 (16.5%)	340 (14.9%)	0.112	286 (16.4%)	252 (14.4%)	0.116
Acute arrhythmia, n (%)	114 (4.0%)	89 (3.9%)	0.830	82 (4.7%)	72 (4.1%)	0.465
Pulmonary embolism, n (%)	21 (0.7%)	47 (2.1%)	< 0.001	14 (0.8%)	29 (1.7%)	0.031
Laboratory results upon admission:						
WBC, count/µL, median [IQR]	9.2 [7.2–12.2]	9.3 [7.2–12.4]	0.703	9.1 [7.2–12.1]	9.3 [7.2–12.4]	0.212
Lymphocytes, count/µL, median [IQR]	1.3 [0.8–1.9]	1.4 [0.9–2.0]	< 0.001	1.3 [0.8–1.8]	1.4 [0.9–2.0]	< 0.001
Neutrophils, count/µL, median [IQR]	6.7 [5.0–9.5]	6.7 [4.9–9.8]	0.946	6.6 [4.9–9.4]	6.7 [4.9–9.8]	0.653
Hemoglobin, g/dL, mean ± SD	12.8 (± 2.3)	11.8 (± 2.0)	< 0.001	12.8 (± 2.3)	11.8 (± 2.0)	< 0.001
Platelets, count/µL, median [IQR]	214 [168–274]	247 [197–310]	< 0.001	213 [167–270]	247 [199–312]	< 0.001
CRP, mg/L, median [IQR]	17 [4–77]	14 [4–58]	0.003	17 [4–77]	13 [4–57]	0.003
Albumin, g/dL, mean ± SD	3.65 (± 0.54)	3.61 (± 0.54)	0.012	3.66 (± 0.54)	3.63 (± 0.55)	0.100
Creatinine, mg/dL, median [IQR]	1.15 [0.89–1.67]	0.96 [0.72–1.39]	< 0.001	1.15 [0.90–1.64]	0.93 [0.71–1.37]	< 0.001
eGFR, mL/min/1.73 m^2^, median [IQR]	67 [42–89]	61 [38–87]	< 0.001	66 [42–89]	64 [40–88]	0.087
Glucose, mg/dL, median [IQR]	160 [123–221]	155 [121–214]	0.102	157 [122–212]	155 [121–214]	0.961
Potassium, mmol/L, median [IQR]	4.30 [4.00–4.70]	4.30 [3.90–4.70]	< 0.001	4.30 [4.00–4.70]	4.30 [3.90–4.70]	0.007
Sodium, mmol/L, median [IQR]	136 [134–139]	137 [133–139]	0.563	137 [134–139]	137 [133–139]	0.554
HbA1c (%), %, median [IQR]^b^	6.9 [6.2–8.1]	6.8 [6.1–8.0]	0.100	6.9 [6.1–8.0]	6.9 [6.1–8.1]	0.841
HbA1c(mmol/mol) median [IQR]^b^	52.0 [43.5–64.0]	49.0 [43.0–61.0]	0.042	50.0 [43.0–62.2]	49.0 [42.0–60.0]	0.206
Troponin, ng/L, median [IQR]^b^	25.0 [13.8–42.6]	21.0 [6.5–38.6]	< 0.001	25.8 [14.9–42.5]	19.3 [6.5–36.0]	< 0.001
LDL cholesterol, mg/dL, median [IQR]	62 [45–85]	73 [52–99]	< 0.001	63 [45–87]	73 [53–99]	< 0.001
HDL cholesterol, mg/dL, median [IQR]	36 [9, 10, 22, 29–41]	42 [33–53]	< 0.001	37 [9, 10, 22, 30–42]	42 [33–52]	< 0.001
TG, mg/dL, median [IQR]	120.0 [88.0–169.0]	123.0 [92.0–172.2]	0.031	118 [86.0–163.0]	126 [94.0–179.0]	< 0.001
Vital signs upon admission						
Systolic BP, mmHg, mean ± SD	138 (± 26)	140 (± 27)	0.004	138 (± 25)	140 (± 27)	0.031
Diastolic BP, mmHg, mean ± SD	75 (± 15)	74 (± 15)	0.006	75 (± 15)	74 (± 15)	0.049
Pulse, beats/min, median [IQR]	82 [71–95]	82 [71–96]	0.992	82 [71–96]	82 [71–95]	0.548
Temperature, °C, median [IQR]	36.8 [36.6–37.1]	36.8 [36.6–37.0]	0.678	36.8 [36.6–37.1]	36.8 [36.6–37.0]	0.651

ACS, acute coronary syndrome; ADHF, acute decompensated heart failure; AKI, acute kidney injury; BP, blood pressure; COPD, chronic obstructive pulmonary disease; CRP, C-reactive protein; CVA, cerebrovascular accident; eGFR, estimated glomerular filtration rate (using CKD-EPI equation); HDL, high density lipoprotein; PVD, peripheral vascular disease; LDL, low density lipoprotein; TG, triglycerides; TIA, transient ischemic stroke; WBC, white blood cells

^a^AKI- acute kidney injury, was estimated as an increase in laboratory creatinine levels by > 0.3 mg/dL,

^b^Troponin and Hba1C levels were available for 60.1% of men and 57.7% of women, and after matching for 60.5% of men and 58% of women and HbA1c levels were available in 16.5% of men and 16.8% of women and after matching in 15.8% of men and 16.9% of women

Glucose levels upon admission were not statistically different between women and men (*p* = 0.102), nor were HbA1c levels (*p* = 0.1, available for only 21.5% and 22.9% of patients, respectively).

### In-hospital glucose control and hospitalization outcomes

**Fig. 1:**
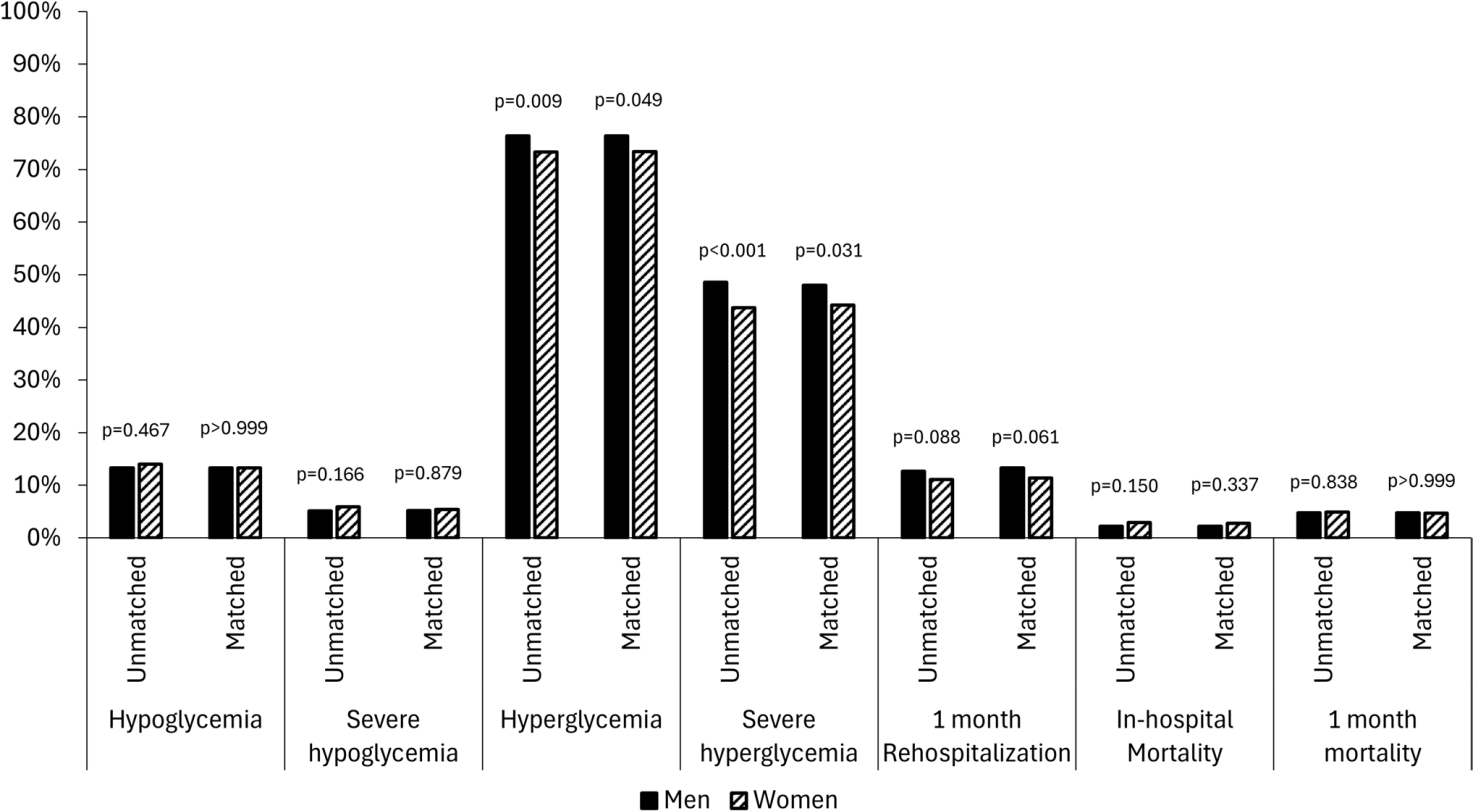
Sex based disparities in the in-hospital glucose control and hospitalization outcomes

During hospitalization, men suffered more often from hyperglycemic episodes (76.4% vs. 73.3%, *p* = 0.009), and severe hyperglycemic episodes (48.6% vs. 43.7%, *p* < 0.001) compared with women. Overall, women had more measurements in range during hospitalization (60% vs. 57.1%, *p* = 0.036). Rehospitalization after 1 month was higher in men – 12.8% vs. 11.1%, though this difference was not statistically significant (*p* = 0.088). There were no differences in mortality outcomes (Table 3; Figure 1).

**Table 3:** In-Hospital glucose control and outcomes before and after matching

	Unmatched cohort			Matched cohort		
	Men (*n* = 2845)	Women (*n* = 2288)	*P*-value	Men (*n* = 1755)	Women (*n* = 1755)	*P*-value
Hypoglycemia^a^, n (%)	378 (13.3%)	320 (14.0%)	0.467	234 (13.3%)	233 (13.3%)	> 0.999
Severe hypoglycemia^a^, n (%)	144 (5.1%)	136 (5.9%)	0.166	91 (5.2%)	94 (5.4%)	0.879
Hyperglycemia^a^, n (%)	2175 (76.4%)	1676 (73.3%)	0.009	1340 (76.4%)	1288 (73.4%)	0.049
Severe hyperglycemia^a^, n (%)	1384 (48.6%)	999 (43.7%)	< 0.001	842 (48.0%)	776 (44.2%)	0.031
Measurements in range^a^ (%)	57.1 [33.3–80.0]	60.0 [36.1–80.0]	0.036	58.3 [35.4–80.0]	60.0 [36.0–80.0]	0.328
Hospitalization Length (days), median [IQR]	5 [4–9]	6 [4–10]	0.123	6 [4–10]	6 [4–9]	0.459
Rehospitalization 1 M, n (%)	353 (12.7%)	247 (11.1%)	0.088	229 (13.3%)	194 (11.4%)	0.061
In hospital Mortality, n (%)	64 (2.2%)	66 (2.9%)	0.150	39 (2.2%)	49 (2.8%)	0.337
1 M mortality, n (%)	137 (4.8%)	113 (4.9%)	0.838	84 (4.8%)	83 (4.7%)	> 0.999

m-*P* value using McNemar after propensity score matching for: BMI, age, gender, steroid intake, COPD asthma, liver disease, chronic infection, HTN, CHF, IHD, Atrial fibrillation flutter, cardiac arrhythmias, PVD, dyslipidemia, past stroke TIA, dementia, and malignancy

^a^Hypoglycemia ≤ 70 mg/dL, severe hypoglycemia ≤ 54 mg/dL, normal glucose values- 71–180 mg/dL, hyperglycemia 181 <, and severe hyperglycemia 250 < mg/dL Range- 100–180 mg/dL, 1M- 1 month

### The association between sex and main hospitalization characteristics and outcomes

Men were treated more often with aspirin both in univariate analysis [OR 1.33 (95%CI 1.18–1.5), *p* < 0.001 and after adjustment in multivariable analysis [aOR 1.31 (95%CI 1.13–1.52) *p* < 0.001]. SGLT treatment was not more common in men after multivariable analysis aOR 1.25 (95%CI (0.94–1.64), *p* = 0.113]. Male sex was associated with hyperglycemia [OR 1.18 (95% CI 1.04–1.34), *p* = 0.008] and severe hyperglycemia [OR 1.22 (95%CI (1.09–1.36 *p* < 0.001)]. It remained independently associated with hyperglycemia [aOR 1.23 (95%CI 1.03–1.46 *p* = 0.02)] and severe hyperglycemia [aOR 1.28 (95% CI (1.09–1.49, *p* = 0.002)] after adjustment in multivariable analysis. 1-month rehospitalization rates tended to be higher in men [OR 1.16 (95% CI 0.97–1.38, *p* = 0.088] in univariate analysis, and became significant after adjustment in multivariable analysis [aOR 1.27 (95%CI 1.03–1.55, *p* = 0.02)] (Table 4).

**Table 4:** The association between male sex and main hospitalization characteristics and outcomes

	OR (95%CI)	*P*-value	aOR^c^ (95%CI)	*P*-value
Chronic medications				
Aspirin	1.33 (1.18–1.50)	< 0.001	1.31 (1.13–1.52)	< 0.001
Plavix	1.59 (1.3–1.93)	< 0.001	1.29 (1.01–1.63)	0.036
Statin	1.15 (1.03–1.28)	0.010	1.10 (0.96–1.26)	0.131
GLP1 agonist	1.06 (0.83–1.37)	0.607	0.82 (0.61–1.11)	0.201
SGLT2 inhibitor	1.6 (1.27–2.02)	< 0.001	1.25 (0.94–1.64)	0.113
Ace inhibitors	1.33 (1.18–1.50)	< 0.001	1.31 (1.13–1.52)	< 0.001
In-hospital glucose control^a^				
Hyperglycemia^a^	1.18 (1.04–1.34)	0.008	1.23 (1.03–1.46)	0.020
Severe hyperglycemia^a^	1.22 (1.09–1.36)	< 0.001	1.28 (1.09–1.49)	0.002
Hypoglycemia^a^	0.94 (0.80–1.10)	0.467	1.11 (0.91–1.34)	0.298
Severe hypoglycemia^a^	0.84 (0.66–1.07)	0.166	1.05 (0.79–1.39)	0.741
Hospitalization outcomes				
Rehospitalization 1 M	1.16 (0.97–1.38)	0.088	1.27 (1.03–1.55)	0.020
In hospital mortality	0.77 (0.54–1.09)	0.151	0.89 (0.60–1.32)	0.558
30-day mortality	0.97 (0.75–1.25)	0.838	1.15 (0.85–1.56)	0.360
Mortality or prolonged stay^b^	0.90 (0.80–0.99)	0.070	1.12 (0.97–1.29)	0.119

ACE, angiotensin converting enzyme; GLP-1, Glucagon-like peptide-1; SGLT2, sodium-glucose transport protein 2

^a^Hypoglycemia ≤ 70 mg/dL, severe hypoglycemia ≤ 54 mg/dL, normal glucose values- 71–180 mg/dL, hyperglycemia 181 <, and severe hyperglycemia 250 < mg/dLRange- 100–180 mg/dL

^b^Prolonged stay-hospitalization > 7 days

M- month

^c^aOR adjusted odds ratio- adjusted for male sex, age and variables with *p* < 0.1 using a stepwise forward regression analysis

### Matched cohorts

#### Study population – matched cohort

The matched cohort consisted of two similar groups, comprising 1,755 men and women (Table 1 and Supplementary Table S3). Men had higher rates of CKD (16.3% vs. 13.2%, *p* = 0.012) and women had higher rates of connective tissue disease (4.8% vs. 1.4%, *p* < 0.001). Men were more frequently treated with aspirin (34.7% vs. 29.3%, *p* < 0.001) and SGLT2 inhibitors (7.7% vs. 5.5%, *p* = 0.013). LDL levels were higher in women (median 73 mg/dL vs. 63 mg/dL, *p* < 0.001) despite similar statin use.

The cause of admission differed between men and women: Acute infection was more common in men than in women (33.3% vs. 29.7%, *p* = 0.019). Chest pain and ACS were also more common in men (16.7% vs. 13.7%, *p* = 0.016, and 24.5% vs. 20.7%, *p* = 0.002, respectively), which was manifested in higher troponin levels in men (25.8 ng/L vs. 19.3 ng/L *p* < 0.001). Women were more often diagnosed with pulmonary embolism (1.7% vs. 0.8%, *p* = 0.031).

#### In-hospital glucose control and hospitalization outcomes

During hospitalization, men suffered more often from hyperglycemic episodes (76.4% vs. 73.4%, *p* = 0.049), and severe hyperglycemic episodes (48% vs. 44.2%, *p* = 0.031) compared with women. Overall, women had more measurements in the desirable range during hospitalization (60% vs. 57.1%, *p* = 0.036). Rehospitalization after 1 month was higher in men (13.3% vs. 11.4%), though this difference was not statistically significant (*p* = 0.061). No significant differences in mortality were observed post-matching (Table 3; Figure 1).

## Discussion

This study highlights sex-based differences in hospitalized patients with T2DM, particularly in baseline cardiovascular risk, chronic treatment patterns, glycemic control, and hospitalization outcomes. Men had a higher burden of cardiovascular disease (IHD, CKD, and PVD) and were more frequently prescribed cardioprotective medications (statins, aspirin, and SGLT2 inhibitors). Ischemic complications were a more common cause of hospitalization in men. Importantly, male sex was independently associated with worse outcomes, including an increased risk of hyperglycemia, severe hyperglycemia, and 1-month rehospitalization.

Hyperglycemia during hospitalization is related to worse outcomes across a range of clinical conditions, in both diabetic and non-diabetic patients [27–29]. However, the interaction between sex and hyperglycemia-related outcomes remains less clearly defined. For example, in patients hospitalized with acute coronary syndrome, admission hyperglycemia was independently associated with increased mortality in men but not in women [30, 31]. Conversely, another study reported that elevated HBA1c levels were associated with rehospitalization in women with CVD but not in men [32]. 

In our study, men experienced a higher frequency of hyperglycemia events during hospitalization. In general, in-hospital treatment protocols do not differ between men and women and typically involve initiating insulin treatment when blood glucose levels are > 180 mg/dL in ≥ 2 measurements, using weight-based dosages. Despite this standardized approach, previous studies suggest that glycemic control may differ between hospitalized men and women with diabetes. For example, a study focusing on anthropometric and body composition measurements found men to have higher blood glucose fluctuations [33]. Sex hormones play a key role in glucose regulation and may contribute to these differences. Testosterone deficiency in older men is related to metabolic syndrome, visceral adiposity and increased insulin resistance [34], while androgen excess in women is related to increased risk of diabetes. Elevated levels of sex hormone-binding globulins appear to have a protective effect, particularly in women [35]. In contrast, a clinical study of critically ill patients reported greater insulin resistance in women than in men, highlighting the variability of sex-based metabolic responses under different physiological conditions [22].

Beyond hospitalization characteristics and outcomes, our study also revealed sex-based differences in chronic treatment, co-morbidities and reasons for acute admission. Notably, men were more likely to receive cardioprotective therapies, such as antiplatelet agents, even after propensity score matching and multivariable adjustment. Previous studies have shown that men with diabetes are more likely than women to receive guideline-directed treatment for cardiovascular risk and complications [7–9].

It is well established that treatment with SGLT2 inhibitors reduces the risk of major cardiovascular events and all-cause mortality in patients with T2DM when added to standard care [36, 37]. Accordingly, current diabetes treatment guidelines recommend SGLT2 as a standard therapy for adults with T2DM and established or high risk of atherosclerotic cardiovascular disease, HF, or CKD [38]. A large meta-analysis, found no significant sex differences in HbA1c reduction or major adverse cardiovascular outcomes (MACE) with either SGLT2 inhibitors or GLP1 agonists [39]. Consistent with previous research, this study demonstrates low overall SGLT2 inhibitors or GLP1 agonists treatment. However, men were more frequently prescribed with SGLT2 inhibitors than women [OR 1.39 (95%CI 1.061–1.828), *p* = 0.0169]. Similar results were reported in a large retrospective study involving 934,737 patients, where women were less likely than men to receive SGLT2 inhibitors; aOR, 0.84; 95% CI, 0.82–0.85 [9].

Statins have the same effectiveness in men and women with similar cardiovascular risk factors [40]. Yet, women used statins less often than men (RR 0.90; 95% CI 0.86, 0.93) [10]. In our study, women had higher LDL levels (*p* < 0.001) and men were more often chronically treated with statins (*p* = 0.011), though this was not significant after matching and multivariable regression.

Women have lower in-hospital mortality rate than men for a wide variety of medical conditions, both infectious [41] and non-communicable diseases [40]. Yet, diabetic women have similar or even higher mortality rates compared to men [42]. This was also demonstrated in our study, though we could not address the causes of death. Treatment disparities may partly explain the lack of observed sex-based differences in mortality.

### Study limitations

This retrospective, large-scale study has inherent limitations due to its observational nature and is subject to residual confounding. We relied on coded data, and it is possible that some information was not recorded. For example, physicians may not have coded all the medications a patient received or all comorbid conditions. Nevertheless, such underreporting is unlikely to differ between men and women. Therefore, if present, it would most likely attenuate the observed association (i.e., non-differential misclassification bias). Glucose levels were assessed using point-of-care measurements rather than continuous glucose monitoring (CGM), which is currently being evaluated in ongoing studies [40]. CGM offers advantages, particularly in detecting hypoglycemia, including nocturnal events, which may differ by sex and are difficult to capture with routine measurements.

[43] Since our study had limited number of glucose measurements, a native-binomial regression model with length of stay in the denominator could have provided additional information. However, since the duration of hospitalization did not differ significantly between men and women, both before and after matching, and given that the current sample size provides high power to detect even small differences in length of stay, we can assume that the occurrence of an event is likely unrelated to the duration of hospitalization.

Additionally, Shamir Medical Center serves a highly heterogeneous population with diverse religious, cultural, and socioeconomic backgrounds. Data on these variables were not systematically available and could not be incorporated into the current analyses. Therefore, potential interactions between these factors and sex on diabetes outcomes could not be evaluated.

Finally, the follow-up was limited to 30 days and different sex related outcomes may appear with longer follow up. Further research should explore sex-specific responses to inpatient glucose management interventions.

## Conclusion

Our findings support the development of hospital protocols that integrate sex-specific glucose monitoring strategies and equitable medication prescribing practices. Since men are more prone to hyperglycemia, they may require closer monitoring and more aggressive insulin adjustment during hospitalization.

In addition, optimizing guideline-directed cardioprotective therapy, especially in women, is crucial. Given the persistent sex gap in statin and SGLT2 inhibitor prescriptions, further efforts are needed to ensure equitable treatment.

## Supplementary Information

Below is the link to the electronic supplementary material.

Supplementary file1 (DOCX 39 KB)

## Data Availability

The datasets generated and analyzed during the current study are not publicly available due to institutional and national data protection regulations. However, aggregated data and analytic code supporting the findings of this study are available from the corresponding author upon reasonable request.

## Code availability

The datasets generated during and/or analysed during the current study are available from the corresponding author on reasonable request.
