# Supplementary material for: Sex based disparities in glycemic control and hospitalization outcomes of medical patients with diabetes mellitus—a historical cohort study
Source: Intern Emerg Med. 2025 Nov 1;21(1):141–51. doi: 10.1007/s11739-025-04170-4 (PMC12948814; doi:10.1007/s11739-025-04170-4)
Supplement: Supplementary file 1 — Supplementary file1 (DOCX 39 KB) [file 11739_2025_4170_MOESM1_ESM.docx]

Supplementary Table 1: ICD-codes used for patients’ diagnosis:

| **CONDITION** | **ICD-9 CODE** |
| --- | --- |
| Chest pain | 786.50, 786.59 |
| Dyspnea | 786.0 |
| Infection | 1-139, 320-326, 420-422, 429, 480-488, 460-466, 510, 519, 567, 569, 575, 601, 604, 576.1, 590.1, 680-686, 711, 730, 780.6, 995.91, 995.92 |
| Ischemic heart disease | 410-414 |
| Congestive heart failure | 428 |
| Atrial fibrillation/flutter | 427.3 |
| Cardiac arrythmia | 426, 427.1, 427.2. 427.4, 427.5, 427.6, 427.8, 427.9 |
| Acute kidney injury | 584, 586 |
| Dementia | 290 |
| Liver disease | 570-573 |
| Connective tissue disease | 710-720, 725 |
| Hypertension | 401 |
| Peripheral vascular disease | 443 |
| Dyslipidemia | 272 |
| Chronic kidney disease | 585-586 |
| Malignancy | 140-239 |
| COPD/Asthma | 490-496 |
| Pulmonary embolism | 415.1 |

COPD- Chronic obstructive pulmonary disease.

Supplementary Table 2:

| **Medication** | **ATC group** |
| --- | --- |
| Metformin | A10BA, A10BD |
| DPP4 | A10BD07, A10BD08, A10BH |
| GLP1 | A10BJ, A10BX04, A10BX07, A10BX08, A10AE56 |
| SU | A10BB |
| Non-SU | A10BX01, A10BX02, A10BX03 |
| Thiazolidinediones | A10BG |
| SGLT2 | A10BK, A10BX09, A10BX10, A10BX11, A10BX12, A10BX13, A10BD15, A10BD16, A10BD20 |
| Insulin basal | A10AE, A10AD, A10AC |
| Insulin Bolus | A10AB, , A10AD |
| Steroids | H02AB |
| Aspirin | B01AC06, N02BA01 |
| Plavix | B01AC04 |
| Statins | C10AA01, C10AA02, C10AA03, C10AA04, C10AA05, C10AA07 |
| Ace inhibitors/ARBs | C09A, C09B, C09C, C09D |

ACE angiotensin converting enzyme, ARB -angiotensin receptor blockers, DPP4- Dipeptidyl peptidase-4, GLP-1 Glucagon-like peptide-1, SU-sulfonylurea, SGLT2- sodium-glucose transport protein 2,

Supplementary Table 3: Standardized differences of thr two sex groups, before and after matching.

|  | Unmatched cohort | | | | Macthed cohort | | | |
| --- | --- | --- | --- | --- | --- | --- | --- | --- |
|  | **MEN (n = 2845)** | **WOMEN (n = 2288)** | **P-Value** | **SMD** | **Men^m^ (n=1755)** | **Women^m^ (n=1755)** | **P-value^m^** | **SMD** |
| Age, years, median [IQR] | 74 [66-81] | 77 [70-85] | <0.001 | 0.268 | 75 [68-82] | 75 [68-83] | 0.243 | 0 |
| BMI, kg/m^2, median [IQR] | 27.3 [24.5-30.9] | 28.1 [24.8-32.4] | <0.001 | 0.153 | 27.7 [24.7-31.2] | 27.7 [24.2-31.6] | 0.981 | 0 |
| **Comorbidities** | | | | | | | | |
| HTN, n (%) | 1337 (47.0%) | 1206 (52.7%) | <0.001 | 0.114 | 881 (50.2%) | 882 (50.3%) | 1 | 0.002 |
| Dyslipidemia, n (%) | 1331 (46.8%) | 1118 (48.9%) | 0.138 | 0.042 | 857 (48.8%) | 839 (47.8%) | 0.555 | 0.02 |
| CHF, n (%) | 280 (9.8%) | 224 (9.8%) | 0.951 | 0 | 159 (9.1%) | 160 (9.1%) | 1 | 0 |
| IHD, n (%) | 758 (26.6%) | 312 (13.6%) | <0.001 | 0.324 | 296 (16.9%) | 277 (15.8%) | 0.297 | 0.03 |
| Atrial fibrillation/flutter, n (%) | 369 (13.0%) | 359 (15.7%) | 0.005 | 0.077 | 230 (13.1%) | 250 (14.2%) | 0.358 | 0.032 |
| Past stroke/TIA, n (%) | 334 (11.7%) | 198 (8.7%) | <0.001 | 0.099 | 167 (9.5%) | 161 (9.2%) | 0.764 | 0.01 |
| CKD, n (%) | 488 (17.2%) | 322 (14.1%) | 0.003 | 0.085 | 286 (16.3%) | 232 (13.2%) | 0.0128 | 0.087 |
| COPD/Asthma, n (%) | 300 (10.5%) | 242 (10.6%) | 0.97 | 0 | 183 (10.4%) | 179 (10.2%) | 0.866 | 0.007 |
| Liver disease, n (%) | 83 (2.9%) | 99 (4.3%) | 0.007 | 0.075 | 61 (3.5%) | 63 (3.6%) | 0.924 | 0.005 |
| Connective tissue disease, n (%) | 36 (1.3%) | 110 (4.8%) | <0.001 | 0.204 | 25 (1.4%) | 85 (4.8%) | <0.001 | 0.196 |
| PVD, n (%) | 143 (5.0%) | 42 (1.8%) | <0.001 | 0.177 | 43 (2.5%) | 40 (2.3%) | 0.812 | 0.013 |
| Dementia, n (%) | 51 (1.8%) | 72 (3.1%) | 0.002 | 0.084 | 40 (2.3%) | 37 (2.1%) | 0.820 | 0.014 |
| Malignancy, n (%) | 260 (9.1%) | 218 (9.5%) | 0.633 | 0.014 | 174 (9.9%) | 169 (9.6%) | 0.819 | 0.01 |
| Charlson Comorbidity Index, median [IQR] | 5 [4-7] | 5 [4-7] | <0.001 | 0 | 5 [4-7] | 5 [4-7] | 0.201 | 0 |
| **Cause of admission** | | | | | | | | |
| Acute infection, n (%) | 956 (33.6%) | 688 (30.1%) | 0.007 | 0.075 | 585 (33.3%) | 521 (29.7%) | 0.019 | 0.078 |
| Chest pain, n (%) | 491 (17.3%) | 294 (12.8%) | <0.001 | 0.126 | 293 (16.7%) | 241 (13.7%) | 0.0164 | 0.084 |
| Dyspnea, n (%) | 273 (9.6%) | 282 (12.3%) | 0.002 | 0.086 | 171 (9.7%) | 205 (11.7%) | 0.071 | 0.065 |
| COPD/Asthma decompensation, n (%) | 326 (11.5%) | 261 (11.4%) | 0.954 | 0.003 | 198 (11.3%) | 193 (11.0%) | 0.829 | 0.01 |
| ADHF, n (%) | 465 (16.3%) | 386 (16.9%) | 0.614 | 0.016 | 285 (16.2%) | 286 (16.3%) | 1 | 0.003 |
| Acute IHD, n (%) | 936 (32.9%) | 420 (18.4%) | <0.001 | 0.332 | 430 (24.5%) | 364 (20.7%) | 0.002 | 0.091 |
| Acute atrial fibrillation/flutter, n (%) | 457 (16.1%) | 442 (19.3%) | 0.002 | 0.084 | 300 (17.1%) | 308 (17.5%) | 0.755 | 0.011 |
| Acute TIA/CVA, n (%) | 489 (17.2%) | 332 (14.5%) | 0.009 | 0.074 | 271 (15.4%) | 248 (14.1%) | 0.295 | 0.037 |
| *AKI, n (%) | 468 (16.5%) | 340 (14.9%) | 0.112 | 0.087 | 286 (16.4%) | 252 (14.4%) | 0.116 | 0.113 |
| Acute arrhythmia, n (%) | 114 (4.0%) | 89 (3.9%) | 0.83 | 0.005 | 82 (4.7%) | 72 (4.1%) | 0.465 | 0.029 |
| Pulmonary embolism, n (%) | 21 (0.7%) | 47 (2.1%) | <0.001 | 0.119 | 14 (0.8%) | 29 (1.7%) | 0.031 | 0.081 |
| **Laboratory results upon admission** | | | | | | | | |
| WBC , count/µL, median [IQR] | 9.2 [7.2-12.2] | 9.3 [7.2-12.4] | 0.703 | 0.026 | 9.1 [7.2-12.1] | 9.3 [7.2-12.4] | 0.212 | 0.053 |
| Lymphocytes, count/µL, median [IQR] | 1.3 [0.8-1.9] | 1.4 [0.9-2.0] | <0.001 | 0.122 | 1.3 [0.8-1.8] | 1.4 [0.9-2.0] | <0.001 | 0.127 |
| Neutrophils, count/µL, median [IQR] | 6.7 [5.0-9.5] | 6.7 [4.9-9.8] | 0.946 | 0 | 6.6 [4.9-9.4] | 6.7 [4.9-9.8] | 0.653 | 0.028 |
| Hemoglobin , g/dL, mean ± SD | 12.8 (±2.3) | 11.8 (±2.0) | <0.001 | 0.464 | 12.8 (±2.3) | 11.8 (±2.0) | <0.001 | 0.464 |
| Platelets, count/µL, median [IQR] | 214 [168-274] | 247 [197-310] | <0.001 | 0.404 | 213 [167-270] | 247 [199-312] | <0.001 | 0.421 |
| CRP, mg/L, median [IQR] | 17 [4-77] | 14 [4-58] | 0.003 | 0.063 | 17 [4-77] | 13 [4-57] | 0.003 | 0.084 |
| Albumin, g/dL, mean ± SD | 3.65 (±0.54) | 3.61 (±0.54) | 0.012 | 0.074 | 3.66 (±0.54) | 3.63 (±0.55) | 0.1 | 0.055 |
| Creatinine , mg/dL, median [IQR] | 1.15 [0.89-1.67] | 0.96 [0.72-1.39] | <0.001 | 0.35 | 1.15 [0.90-1.64] | 0.93 [0.71-1.37] | <0.001 | 0.42 |
| eGFR , mL/min/1.73m², median [IQR] | 67 [42-89] | 61 [38-87] | <0.001 | 0.167 | 66 [42-89] | 64 [40-88] | 0.087 | 0.056 |
| Glucose , mg/dL, median [IQR] | 160 [123-221] | 155 [121-214] | 0.102 | 0.07 | 157 [122-212] | 155 [121-214] | 0.961 | 0.029 |
| Potassium , mmol/L, median [IQR] | 4.30 [4.00-4.70] | 4.30 [3.90-4.70] | <0.001 | 0 | 4.30 [4.00-4.70] | 4.30 [3.90-4.70] | 0.007 | 0 |
| Sodium , mmol/L, median [IQR] | 136 [134-139] | 137 [133-139] | 0.563 | 0.243 | 137 [134-139] | 137 [133-139] | 0.554 | 0 |
| HbA1c (%), %, median [IQR] # | 6.9 [6.2-8.1] | 6.8 [6.1-8.0] | 0.1 | 0.071 | 6.9 [6.1-8.0] | 6.9 [6.1-8.1] | 0.841 | 0 |
| HbA1c (mmol/mol), mmol/mol, median [IQR]# | 52.0 [43.5-64.0] | 49.0 [43.0-61.0] | 0.042 | 0.208 | 50.0 [43.0-62.2] | 49.0 [42.0-60.0] | 0.206 | 0.072 |
| Troponin , ng/L, median [IQR]# | 25.0 [13.8-42.6] | 21.0 [6.5-38.6] | <0.001 | 0.176 | 25.8 [14.9-42.5] | 19.3 [6.5-36.0] | <0.001 | 0.305 |
| LDL cholesterol , mg/dL, median [IQR] | 62 [45-85] | 73 [52-99] | <0.001 | 0.338 | 63 [45-87] | 73 [53-99] | <0.001 | 0.304 |
| HDL cholesterol , mg/dL, median [IQR] | 36 [29-44] | 42 [33-53] | <0.001 | 0.455 | 37 [30-45] | 42 [33-52] | <0.001 | 0.391 |
| Triglycerides , mg/dL, median [IQR] | 120.0 [88.0-169.0] | 123.0 [92.0-172.2] | 0.031 | 0.05 | 118 [86.0-163.0] | 126 [94.0-179.0] | <0.001 | 0.132 |
| **Vital signs upon admission** | | | | | | | | |
| Systolic BP , mmHg, mean ± SD | 138 (±26) | 140 (±27) | 0.004 | 0.075 | 138 (±25) | 140 (±27) | 0.031 | 0.077 |
| Diastolic BP , mmHg, mean ± SD | 75 (±15) | 74 (±15) | 0.006 | 0.067 | 75 (±15) | 74 (±15) | 0.049 | 0.067 |
| Pulse , beats/min, median [IQR] | 82 [71-95] | 82 [71-96] | 0.992 | 0 | 82 [71-96] | 82 [71-95] | 0.548 | 0 |
| Temperature , °C, median [IQR] | 36.8 [36.6-37.1] | 36.8 [36.6-37.0] | 0.678 | 0 | 36.8 [36.6-37.1] | 36.8 [36.6-37.0] | 0.651 | 0 |
| **Chronic medication** | | | | | | | | |
| Metformin, n (%) | 1257 (44.2%) | 1043 (45.6%) | 0.315 | 0.028 | 787 (44.8%) | 814 (46.4%) | 0.36 | 0.032 |
| Long-acting Insulin, n (%) | 379 (13.3%) | 281 (12.3%) | 0.268 | 0.03 | 229 (13.0%) | 210 (12.0%) | 0.332 | 0.03 |
| Short-acting Insulin, n (%) | 153 (5.4%) | 116 (5.1%) | 0.623 | 0.013 | 79 (4.5%) | 91 (5.2%) | 0.345 | 0.053 |
| SU, n (%) | 141 (5.0%) | 87 (3.8%) | 0.046 | 0.059 | 89 (5.1%) | 70 (4.0%) | 0.123 | 0.057 |
| Non-SU, n (%) | 151 (5.3%) | 100 (4.4%) | 0.122 | 0.042 | 96 (5.5%) | 65 (3.7%) | 0.012 | 0.086 |
| DPP4, n (%) | 154 (5.4%) | 143 (6.2%) | 0.202 | 0.034 | 95 (5.4%) | 102 (5.8%) | 0.608 | 0.017 |
| GLP1 agonists, n (%) | 147 (5.2%) | 111 (4.9%) | 0.607 | 0.014 | 100 (5.7%) | 95 (5.4%) | 0.761 | 0.013 |
| SGLT2 inhibitor,, n (%) | 223 (7.8%) | 115 (5.0%) | <0.001 | 0.114 | 135 (7.7%) | 97 (5.5%) | 0.013 | 0.089 |
| Any BP Medication, n (%) | 1181 (41.5%) | 1022 (44.7%) | 0.023 | 0.065 | 734 (41.8%) | 745 (42.5%) | 0.707 | 0.014 |
| Ace inhibitors, n (%) | 1310 (46.0%) | 1091 (47.7%) | 0.242 | 0.034 | 831 (47.4%) | 814 (46.4%) | 0.581 | 0.02 |
| Plavix, n (%) | 313 (11.0%) | 165 (7.2%) | <0.001 | 0.132 | 168 (9.6%) | 136 (7.7%) | 0.059 | 0.068 |
| Aspirin, n (%) | 1019 (35.8%) | 674 (29.5%) | <0.001 | 0.134 | 609 (34.7%) | 515 (29.3%) | <0.001 | 0.116 |
| Statins, n (%) | 1532 (53.8%) | 1150 (50.3%) | 0.011 | 0.07 | 931 (53.0%) | 879 (50.1%) | 0.085 | 0.058 |
| Thiazolidinediones, n (%) | 36 (1.3%) | 31 (1.4%) | 0.779 | 0.009 | 20 (1.1%) | 27 (1.5%) | 0.304 | 0.035 |
| Diuretics, n (%) | 404 (14.2%) | 394 (17.2%) | 0.003 | 0.082 | 253 (14.4%) | 278 (15.8%) | 0.239 | 0.039 |
| Systemic Steroids, n (%) | 271 (9.5%) | 231 (10.1%) | 0.494 | 0.02 | 182 (10.4%) | 170 (9.7%) | 0.5 | 0.023 |
| Hospitalization long-acting Insulin, n (%) | 1137 (40.0%) | 809 (35.4%) | <0.001 | 0.095 | 697 (39.7%) | 639 (36.4%) | 0.044 | 0.068 |
| Hospitalization short-acting Insulin, n (%) | 1066 (37.5%) | 815 (35.6%) | 0.172 | 0.039 | 657 (37.4%) | 656 (37.4%) | 0.972 | 0 |

ACE angiotensin converting enzyme, ACS- acute coronary syndrome, ADHF- acute decompensated heart failure, AKI- acute kidney injury, ARB -angiotensin receptor blockers, CHF- congestive heart failure, BMI- body mass index, BP-blood pressure, CKD chronic kidney disease, COPD- chronic obstructive pulmonary disease, CRP- C-reactive protein, CVA- cerebrovascular accident, DPP4- Dipeptidyl peptidase-4 , eGFR- estimated glomerular filtration rate (using CKD-EPI equation), GLP-1 Glucagon-like peptide-1, HDL- high density lipoprotein, HTN- hypertension, IHD- ischemic heart disease, PVD- peripheral vascular disease, LDL- low density lipoprotein, SGLT2- sodium-glucose transport protein 2 TG-triglycerides, SMD- standardized mean difference, SU-sulfonylurea, TIA- transient ischemic stroke, WBC- white blood cells

*AKI- acute kidney injury, was estimated as an increase in laboratory creatinine levels by >0.3 mg/dL,

#Troponin and Hba1C levels were available for 60.1% of men and 57.7% of women, and after matching for 60.5% of men and 58% of women and HbA1c levels were available in 16.5% of men and 16.8% of women and after matching in 15.8% of men and 16.9% of women.
